# An estimate on the Bedrosian commutator in Sobolev space

**DOI:** 10.1186/s13660-018-1940-3

**Published:** 2018-12-17

**Authors:** Marcel Oliver

**Affiliations:** School of Engineering and Science, Jacobs University, Bremen, Germany

**Keywords:** 26D10, 42A85, 46E35, Bedrosian identity, Sobolev space, Banach algebra

## Abstract

While the Bedrosian identity for the Hilbert transform of a product does not hold for general Sobolev class functions, we show that the defect of this identity is more regular than would be naively expected. We use this result to give a stronger-than-expected estimate on the chain rule defect of the square root of the Laplacian.

## Introduction

In 1963, Bedrosian [2] proved the following result on the Hilbert transform of the product of two functions: Let $u, v \in L ^{2}(\mathbb {R})$ satisfy either $\operatorname{supp}\hat{u} \subset[0,\infty)$ and $\operatorname{supp}\hat{v} \subset[0,\infty)$ or $\operatorname{supp}\hat{u} \subset[-a,a]$ and $\operatorname{supp}\hat{v} \cap[-a,a] = \emptyset$ for some $a>0$, then

1 $$ H(uv) = u Hv . $$

This result is now known as the *Bedrosian theorem* and (1) as the *Bedrosian identity*. Here and henceforth, following standard notation, we write

2 $$ \hat{f}(\xi) = \frac{1}{2 \pi} \int_{\mathbb {R}}\mathrm {e}^{-\mathrm {i}\xi x} f(x) \,\mathrm{d}x $$

to denote the Fourier transform of a function $f \in L^{2}(\mathbb {R})$ and *Hf* to denote its Hilbert transform, implicitly defined for a.e. $\xi \in \mathbb {R}$ via

3 $$ (Hf)\hat{\,}(\xi) = -\mathrm {i}\operatorname{sign}\xi\hat{f}(\xi). $$

The Bedrosian identity usually arises in the context of time-frequency analysis. A key question there is the notion of instantaneous amplitude and frequency, where the instantaneous frequency is the derivative of the phase function and is required to be non-negative. Signals with this property are called mono-components; arbitrary signals may be decomposed into mono-components in various ways. When the phase signal is already a mono-component, then its product with an amplitude function is also a mono-component if and only if the Bedrosian identity with *u* as amplitude and *v* as phase function holds [5, 6]. This observation led to a quest to characterize necessary and sufficient conditions for the Bedrosian identity [3, 9, 10]. A full characterization of mono-components, which includes mono-components of non-Bedrosian type, is given in [7]. For computational approaches on signal decompositions into mono-components, see, e.g., [4, 8].

In this note, we are asking a very different question. Given that the conditions under which the Bedrosian identity holds are highly non-generic, is it possible to provide a generic estimate on the defect that is stronger than separate estimates on the left- and right-hand sides of (1)? In the following, we shall show that this is indeed the case when estimating the defect—the Bedrosian commutator—in Sobolev space. In the next section, we state and prove this surprisingly simple result. In the final Sect. 3, we show that this result implies that the square root of the Laplacian on $\mathbb {R}$ satisfies the usual chain rule with a defect that is smoother than each of the separate terms.

## The commutator estimate

As usual, we define the Sobolev space $H^{s}(\mathbb {R})$ as the class of functions in $L^{2}(\mathbb {R})$ such that

4 $$ \lVert f \rVert_{H^{s}} = \int_{\mathbb {R}}\bigl(1 + \lvert\xi\rvert\bigr)^{2s} \bigl\lvert \hat{f}(\xi) \bigr\rvert ^{2} \,\mathrm{d}\xi $$

is finite. We now state our result.

### Theorem 1

*Let* $u\in H^{s}(\mathbb {R})$ *for* $s \geq0$ *and* $v \in H^{r}(\mathbb {R})$ *for* $r>\frac{1}{2}$. *Then the Bedrosian commutator*

5 $$ w = u Hv - H(u v) $$

*is also of class* $H^{s}(\mathbb {R})$, *and there exists a constant* *c* *which depends only on* *r* *such that*

6 $$ \lVert w \rVert_{H^{s}} \leq c \lVert u \rVert_{H^{s}} \lVert v \rVert_{H^{r}} . $$

### Proof

By the Fourier convolution theorem, for a.e. $\xi\in \mathbb {R}$,

7 $$\begin{aligned} \hat{w} (\xi) & = \mathrm {i}\operatorname{sign}\xi \int_{\mathbb {R}}\hat{u} (\xi-\eta) \hat{v}(\eta) \,\mathrm{d}\eta- \mathrm {i}\int_{\mathbb {R}}\hat{u} (\xi-\eta) \operatorname{sign}\eta\hat{v}(\eta) \,\mathrm {d}\eta \\ & = 2 \mathrm {i}\chi_{\xi>0}^{ \vphantom{1}} \int_{-\infty}^{0} \hat{u} (\xi-\eta) \hat{v}( \eta) \,\mathrm{d} \eta- 2 \mathrm {i}\chi_{\xi< 0}^{ \vphantom{1}} \int_{0}^{\infty}\hat{u} (\xi-\eta) \hat{v}( \eta) \,\mathrm{d} \eta. \end{aligned}$$

Thus,

8 $$\begin{aligned} \lVert w \rVert_{H^{s}}^{2} & = 4 \int_{0}^{\infty}\bigl(1 + \lvert\xi\rvert \bigr)^{2s} \biggl\vert \int_{0}^{\infty}\hat{u} (-\xi-\eta) \hat{v}(\eta) \,\mathrm{d} \eta\biggr\vert ^{2} \,\mathrm{d}\xi \\ & \quad{} + 4 \int_{0}^{\infty}\bigl(1 + \lvert\xi\rvert \bigr)^{2s} \biggl\vert \int_{0}^{\infty}\hat{u} (\xi+\eta) \hat{v}(-\eta) \,\mathrm{d} \eta\biggr\vert ^{2} \,\mathrm{d}\xi \\ & \equiv4 (I_{1} + I_{2}) , \end{aligned}$$

where we have made the change of variables $\xi\mapsto-\xi$ in the first term and $\eta\mapsto-\eta$ in the second. Each of the right-hand integrals can how be estimated in the same way; we only write out the argument for $I_{1}$. Noting that *ξ* and *η* are non-negative,

9 $$\begin{aligned} I_{1} & \leq \int_{0}^{\infty} \biggl( \int_{0}^{\infty}\bigl(1 + \lvert\xi+ \eta\rvert \bigr)^{s} \bigl\lvert \hat{u} (-\xi-\eta) \bigr\rvert \bigl\lvert \hat{v}(\eta) \bigr\rvert \,\mathrm{d}\eta\biggr)^{2} \,\mathrm {d}\xi \\ & = \int_{0}^{\infty}\bigl\lvert \hat{v}(\eta) \bigr\rvert \int_{0}^{\infty }\bigl\lvert \hat{v}(\zeta) \bigr\rvert \\ & \quad{} \times \int_{0}^{\infty}\bigl(1 + \lvert\xi+ \eta\rvert \bigr)^{s} \bigl\lvert \hat{u} (-\xi-\eta) \bigr\rvert \bigl(1 + \lvert\xi+ \zeta\rvert\bigr)^{s} \bigl\lvert \hat{u} (-\xi -\zeta) \bigr\rvert \,\mathrm{d}\xi\,\mathrm{d}\zeta\,\mathrm{d}\eta \\ & \leq\lVert u \rVert_{H^{s}}^{2} \biggl( \int_{0}^{\infty} \bigl\lvert \hat{v}(\eta) \bigr\rvert \,\mathrm{d}\eta\biggr)^{2}, \end{aligned}$$

where we have used the Fubini theorem in the second step and the Cauchy–Schwarz inequality in the third.

Finally,

10 $$\begin{aligned} \int_{0}^{\infty}\bigl\lvert \hat{v}(\eta) \bigr\rvert \,\mathrm{d} \eta& \leq \int_{0}^{\infty}(1+\eta)^{-r} (1+\eta )^{r} \bigl\lvert \hat{v}( \eta) \bigr\rvert \,\mathrm{d}\eta \\ & \leq\biggl( \int_{0}^{\infty}(1+\eta)^{-2r} \,\mathrm{d}\eta \biggr)^{ \frac{1}{2}} \lVert v \rVert_{H^{r}} , \end{aligned}$$

where the left-hand integral converges whenever $r>\frac{1}{2}$. Inserting (9) and (10) back into (8) completes the proof. □

### Remark

Note that separate Banach algebra estimates (e.g., [1, Theorem 4.39]) on each of the terms of the commutator yield

11 $$ \lVert w \rVert_{H^{s}} \leq c \lVert u \rVert_{H^{s}} \lVert v \rVert_{H^{s}} $$

for $s>\frac{1}{2}$. Thus, Theorem 1 is stronger in that the regularity requirements on *u* and *v* are independent of each other and the embedding barrier $\frac{1}{2}$ applies only to *v*.

## Application

The commutator estimate can be used to study properties of the square root of the Laplacian (defined with the sign convention as a positive operator), i.e., the operator *A* defined via

12 $$ (Af)\hat{\,}(\xi) = \lvert\xi\rvert\hat{f}(\xi) , $$

so that $A = H \partial_{x}$. This operator has the same mapping properties as the derivative operator between Sobolev spaces, but it does not satisfy a product or chain rule. Indeed,

13 $$ A f(g) = H \partial_{x} f(g) = H \bigl(f'(g) g'\bigr) = f'(g) Hg' - w = f'(g) Ag - w . $$

However, applying Theorem 1 with $u=f'(g)$, $v=g'$, and $s=r>\frac{3}{2}$ shows that the chain rule defect *w* is of class $H^{s}(\mathbb {R})$ when *f* is smooth and $g \in H^{s}(\mathbb {R})$, even though the left-hand side of (13) is generally only of class $H^{s-1}(\mathbb {R})$.
